# Testing oxygenated microbubbles via intraperitoneal and intrathoracic routes on a large pig model of LPS‐induced acute respiratory distress syndrome

**DOI:** 10.14814/phy2.15451

**Published:** 2022-09-06

**Authors:** Riaz Ur Rehman Mohammed, Nathaniel T. Zollinger, Andrea R. McCain, Roser Romaguera‐Matas, Seth P. Harris, Keely L. Buesing, Mark A. Borden, Benjamin S. Terry

**Affiliations:** ^1^ Biomedical Engineering Program, Department of Mechanical and Material Engineering University of Nebraska‐Lincoln Lincoln Nebraska USA; ^2^ Institutional Animal Care Program, Office of Research & Economic Development University of Nebraska – Lincoln Lincoln Nebraska USA; ^3^ School of Veterinary Medicine and Biomedical Sciences University of Nebraska – Lincoln Institute of Agriculture and Natural Resources Lincoln Nebraska USA; ^4^ Department of Surgery University of Nebraska Medical Center Omaha Nebraska USA; ^5^ Biomedical Engineering Program University of Colorado Boulder Colorado USA; ^6^ Department of Mechanical Engineering Brigham Young University Provo Utah USA

**Keywords:** acute respiratory distress syndrome, Berlin criteria, intraperitoneal, intrathoracic, oxygenated microbubbles, peripheral oxygenation

## Abstract

With a mortality rate of 46% before the onset of COVID‐19, acute respiratory distress syndrome (ARDS) affected 200,000 people in the US, causing 75,000 deaths. Mortality rates in COVID‐19 ARDS patients are currently at 39%. Extrapulmonary support for ARDS aims to supplement mechanical ventilation by providing life‐sustaining oxygen to the patient. A new rapid‐onset, human‐sized pig ARDS model in a porcine intensive care unit (ICU) was developed. The pigs were nebulized intratracheally with a high dose (4 mg/kg) of the endotoxin lipopolysaccharide (LPS) over a 2 h duration to induce rapid‐onset moderate‐to‐severe ARDS. They were then catheterized to monitor vitals and to evaluate the therapeutic effect of oxygenated microbubble (OMB) therapy delivered by intrathoracic (IT) or intraperitoneal (IP) administration. Post‐LPS administration, the PaO_2_ value dropped below 70 mmHg, the PaO_2_/FiO_2_ ratio dropped below 200 mmHg, and the heart rate increased, indicating rapidly developing (within 4 h) moderate‐to‐severe ARDS with tachycardia. The SpO_2_ and PaO_2_ of these LPS‐injured pigs did not show significant improvement after OMB administration, as they did in our previous studies of the therapy on small animal models of ARDS injury. Furthermore, pigs receiving OMB or saline infusions had slightly lower survival than their ARDS counterparts. The OMB administration did not induce a statistically significant or clinically relevant therapeutic effect in this model; instead, both saline and OMB infusion appeared to lower survival rates slightly. This result is significant because it contradicts positive results from our previous small animal studies and places a limit on the efficacy of such treatments for larger animals under more severe respiratory distress. While OMB did not prove efficacious in this rapid‐onset ARDS pig model, it may retain potential as a novel therapy for the usual presentation of ARDS in humans, which develops and progresses over days to weeks.

## Graphical abstract

Lipid oxygenated microbubbles have been tested on a large pig model of ARDS.

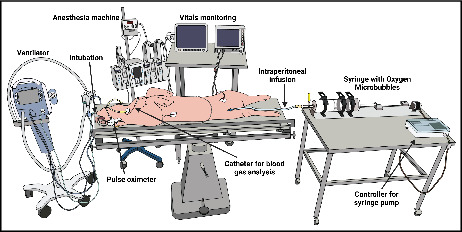

## 1 INTRODUCTION

Acute respiratory distress syndrome (ARDS) is a life‐threatening condition that causes severe respiratory failure. With a pre‐COVID‐19 patient mortality rate of 46%, severe ARDS is known to affect roughly 200,000 people in the US alone, causing 75,000 deaths annually (Bellani et al., 2016). ARDS is one of the principal causes of COVID‐19‐related deaths due to the SARS‐CoV‐2‐mediated cytokine storm (Ragab et al., 2020), and an estimated 33% of hospitalized COVID‐19 patients develop ARDS (Tzotzos et al., 2020). In a recent meta‐analysis reviewing mortality rates in COVID‐19 patients, the overall pooled mortality was found to be 39% in a total of 10,815 ARDS cases (Hasan et al., 2020), which is similar to the non‐COVID‐19 cases of ARDS (Tzotzos et al., 2020). The term ‘ARDS’ was first put forward by (Ashbaugh et al., 1967), based on their observation of patients displaying a clinical, physiological, and pathological course of events similar to the infant respiratory distress syndrome. Since then, revisions have been made to the clinical criteria required for ARDS classification; currently, the Berlin definition of ARDS is widely accepted (Ranieri et al., 2012).

ARDS advances via phases: in the exudative phase, the lung responds to an injury via the innate immune cells. These cells damage the alveolar endothelium and epithelial barrier leading to the accumulation of fluid (edema), followed by a release of inflammatory cytokines. The extent of neutrophil migration during the exudative phase greatly influences the severity of ARDS in the lungs. In the proliferative phase, initiation of repair processes leads to reabsorption of alveolar edema and re‐establishment of the epithelium. In the final fibrotic phase, extensive damage to the basement membrane hinders re‐epithelialization. The final phase does not occur in all individuals suffering from ARDS (Mason et al., 2016). Our study focuses on oxygen status during the early stages of the acute inflammatory (exudative) phase.

Current strategies to mitigate respiratory failure include prone‐positioning, mechanical ventilation, and extracorporeal membrane oxygenation (ECMO; Combes et al., 2018). ECMO works by employing a gas‐exchange device that oxygenates the blood and removes carbon dioxide via a semipermeable membrane (Parekh et al., 2018). Further, ECMO has two configurations: venovenous (deoxygenated blood is drawn from a central vein and reinfused in the central vein) and venoarterial (deoxygenated blood is drawn from a central vein and reinfused in a central artery; Parekh et al., 2018). While ECMO is administered, specific ventilator strategies are recommended (Parekh et al., 2018). Similarly, ventilator‐induced lung injury is another drawback of mechanical ventilation during ARDS (Peek et al., 2010). Prone‐positioning was initially recognized as a salvage therapy to improve oxygenation, but it must be administered as a combination therapy (Lindén et al., 2009). Therefore, oxygenation strategies other than those explained above need to be considered. Treatment goals for all modalities are aimed at limiting damage to the lung parenchyma. Several pathway‐specific immunomodulators for the treatment of ARDS are currently under clinical trials (Slutsky & Ranieri, 2013). In addition, therapies targeting epithelial/endothelial dysfunction are currently being tested to determine their clinical efficacy (Scholten et al., 2017). Despite decades of research, a stand‐alone therapy to cure ARDS has not been found (Combes et al., 2018).

Animal models of ARDS are helpful for the development and evaluation of new therapies. Current animal models for ARDS utilize surfactant lavage, oleic acid administration, smoke inhalation, endotoxin lipopolysaccharide (LPS), and other agents (Hochhausen et al., 2019; Horie et al., 2020; Leiphrakpam, Weber, Ogun, & Buesing, 2021; Lutz et al., 1998; Mehta et al., 2021; Schmidhammer et al., 2006; Stenlo et al., 2020; Zadeh et al., 2019). These models offer different advantages and disadvantages. For example, the lavage model is easily reversible, whereas the LPS model is not. The dose and administration method of LPS and the choice of study animal can also profoundly affect the time course for the development and severity of ARDS (Leiphrakpam, Weber, McCain, et al., 2021; Pompe et al., 1996). This study presents a rapid‐onset lung injury model marked by hypoxemia and tachycardia for evaluating oxygenation therapies, such as the OMB therapy discussed here.

LPS is part of the outer membrane of gram‐negative bacteria and can be administered directly into the airways or systemically (extrapulmonary; Pompe et al., 1996). The time window used for induction and assessment of lung injury varies in the currently described models of LPS‐induced lung injury. Direct LPS‐induced lung injury causes acute and vigorous migration of inflammatory cells into the lung tissue (Brooks et al., 2020). Several LPS‐induced porcine lung injury models have been tested in the past with different levels of complexity. Lutz et al. tested the efficacy of aerosolized surfactant replacement in an LPS‐injured pig model of severe ARDS. The replacement of aerosolized surfactant improved the oxygenation in the porcine model by decreasing leukocyte sequestration (Mehta et al., 2021). Xu et al. showed the blunting effect of a resin‐directed hemoadsorption on an LPS‐induced cytokine storm in a porcine model of ARDS. The resin treatment was found to improve oxygenation in pigs (Domscheit et al., 2020). Furthermore, research studies by Stenlo et al. and Ruemmler et al. also used porcine models of LPS‐induced ARDS to study critical parameters of ARDS (Bozinovski et al., 2004; Lutz et al., 1998). Leiphrakpam, Weber, Ogun, & Buesing (2021) recently reported smoke inhalation‐induced rat and pig models of ARDS (Buesing et al., 2019). This model may be helpful in studying the pathophysiology of inhalation‐induced injury without confounding variables like a subcutaneous injury.

OMBs are colloids consisting of 0.1–10 μm oxygen gas core bubbles in saline coated with a shell made of lipid (Ruemmler et al., 2021; Xu et al., 2017). Previously, we used a rat model of lung trauma (pneumothorax) to test the effect of OMB and oxygenated saline. The pneumothorax model was chosen to examine oxygenation in direct lung injury (Swanson et al., 2010). In that study, rats treated with OMB had an SaO_2_ between 80% and 90%, whereas those administered salines showed a rapid decline in the first 20 min (Swanson & Borden, 2010). The OMB infusion significantly increased the survival times of rats compared with saline: peritoneal infusion of OMB produced 100% survival at the 2 h study endpoint compared with 100% mortality at only 18.5 and 15.5 min in saline and untreated controls, respectively (Swanson & Borden, 2010). In a similar study, we showed that peritoneal membrane oxygenation using OMB doubled the survival time of rabbits experiencing complete tracheal occlusion (Matsutani et al., 2010). The rabbits in the control group (administered saline) had a mean survival time of 6.9 ± 0.6 min, whereas those in the OMB group had a higher survival time of 12.5 ± 3.0 min. Similarly, the average SpO_2_ was found to be greater in rabbits from the OMB group. In another study, a rat model of LPS‐induced ARDS was developed to test the peritoneal infusion of OMB (Feshitan et al., 2014). The rats treated with OMB had a significantly greater SpO_2_ compared with the untreated and saline groups. Moreover, we showed that rats experiencing LPS‐induced moderate ARDS had a 37% greater survival rate after a single bolus of OMB was administered to the peritoneal cavity (Legband et al., 2015). Rodents have lesser sensitivity to endotoxemia than other species, such as pigs (Fiala et al., 2020). These results encouraged us to investigate the effect of OMB on a human‐sized pig model of rapidly developing ARDS. In addition, due to the large mortality rate from ARDS, there is a clear rationale to explore new therapeutic modalities that can supplement oxygenation levels without damaging the lung parenchyma.

For this study, “intraperitoneal” has been abbreviated as ‘IA’ since it has been synonymously used with ‘intraabdominal’. In addition to testing the traditional route of OMB administration via intraperitoneal (IA) infusion, we investigated an additional intrathoracic (IT) OMB infusion modality. The IT route was chosen since the pleural cavity has a prominent vasculature that can facilitate oxygen diffusion into the body's blood circulation. This additional route, if successful, would increase options of administration under diverse clinical scenarios. Therefore, in the present study, we tested the hypotheses that OMB administration via IT or IA catheter increases blood oxygenation in human‐sized pigs suffering from rapid‐onset, LPS‐induced, moderate‐to‐severe ARDS with tachycardia.

## 2 MATERIALS AND METHODS

**FIGURE 1:**
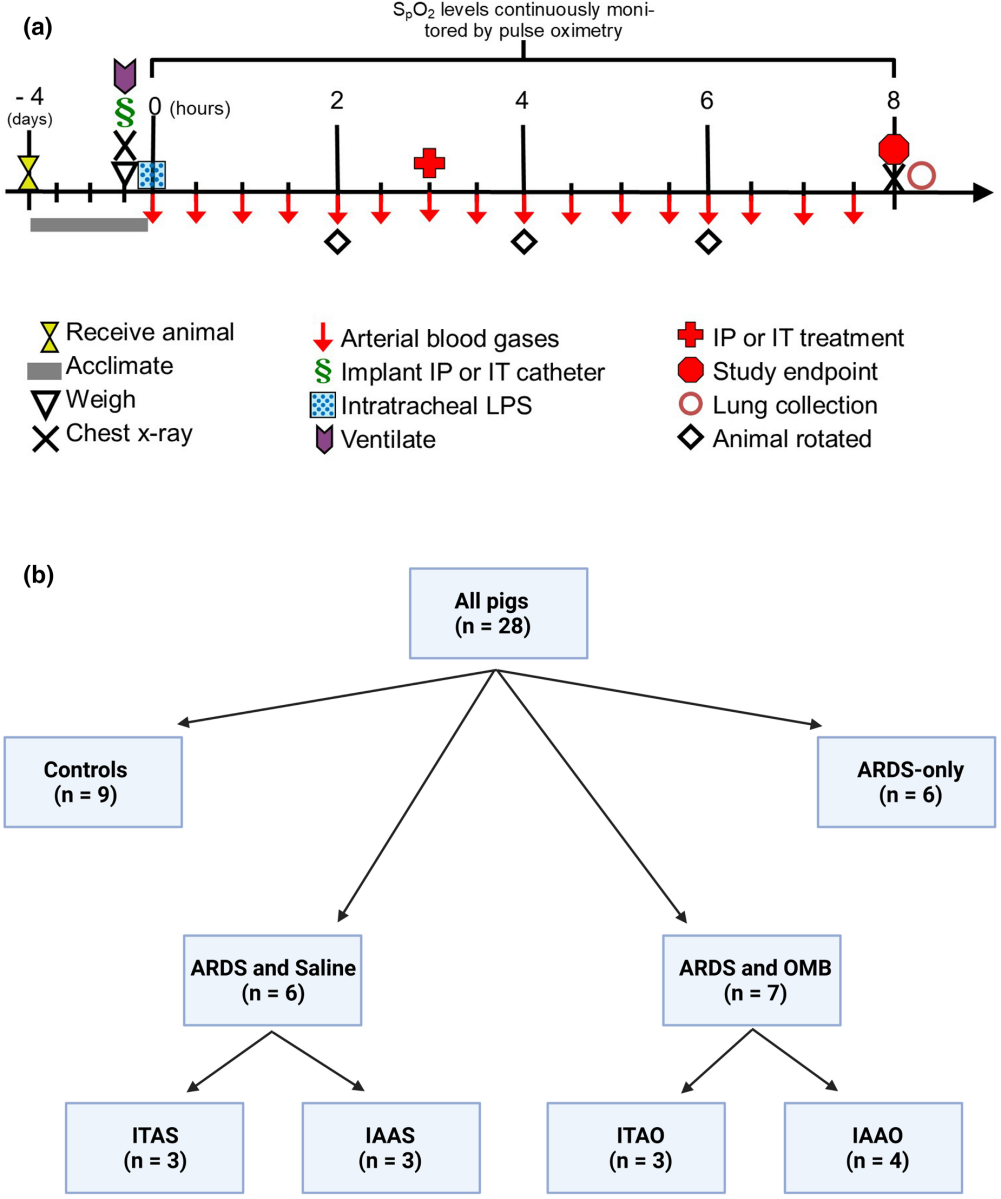
Study information. (a) Experimental timeline. (b) Grouping of pigs for the study

All animal experiments for this study were performed by the University of Nebraska‐Lincoln Animal Care and Use Committee (IACUC) and the Animal Care and Use Review Office (ACURO). The objective of the methodology followed in the experiments was to establish a rapid‐onset (<4 h) moderate‐to‐severe ARDS (PaO_2_/FiO_2_ < 200 mmHg or SpO_2_ < 80% for 30 min) pig model with ~50% mortality rate at 8 h following LPS administration. We chose the direct application of LPS via nebulization at a relatively high dose (4 mg/kg) to achieve this end. This dosage of LPS is greater than prior studies and was necessary to establish a rapid onset of moderate ARDS to meet the required oxygen deficiency criterion to achieve moderate‐to‐severe ARDS within 4 h (Mehta et al., 2021)–(Stenlo et al., 2020). All the experiments were performed according to the timeline shown in Figure 1a.

### 2.1 Grouping of animals

In this study, 28 female Duroc pigs weighing 45 ± 5 kg were purchased from DNA genetics, Waldo genetics, or Plymouth Ag group. All pigs were acclimated to the animal facility for 4 days with free access to food and water. The pigs were randomly divided into (1) a control group (*n* = 9) of healthy pigs that were sedated, catheterized, and ventilated in an animal ICU; (2) an ARDS‐only group (*n* = 6) that were also sedated, catheterized, and ventilated in the animal ICU; (3) an ARDS with saline administration group (*n* = 7); and (4) an ARDS with the OMB administration group (*n* = 6). The ARDS with saline administration group was subdivided into an IT‐ARDS‐saline catheter group (ITAS, *n* = 3) and an IA‐ARDS‐saline catheter group (IAAS, *n* = 3). Similarly, the ARDS with the OMB administration group was subdivided into an IT‐ARDS‐OMB (ITAO, *n* = 3) and IA‐ARDS‐OMB catheter groups (IAAO, *n* = 4). Note that the IAAO group had one more pig than the others simply due to the fact that pigs assigned to this group experienced one less random event that caused dropouts. In total, the pigs were organized into six study groups in the manner mentioned above to test the hypotheses that an ICU pig with rapid‐onset moderate‐to‐severe ARDS experiences a clinically relevant rise in oxygenation after receiving an OMB infusion via an IT or IA catheter (Figure 1b).

### 2.2 Setting up a porcine intensive care unit (ICU)

**FIGURE 2:**
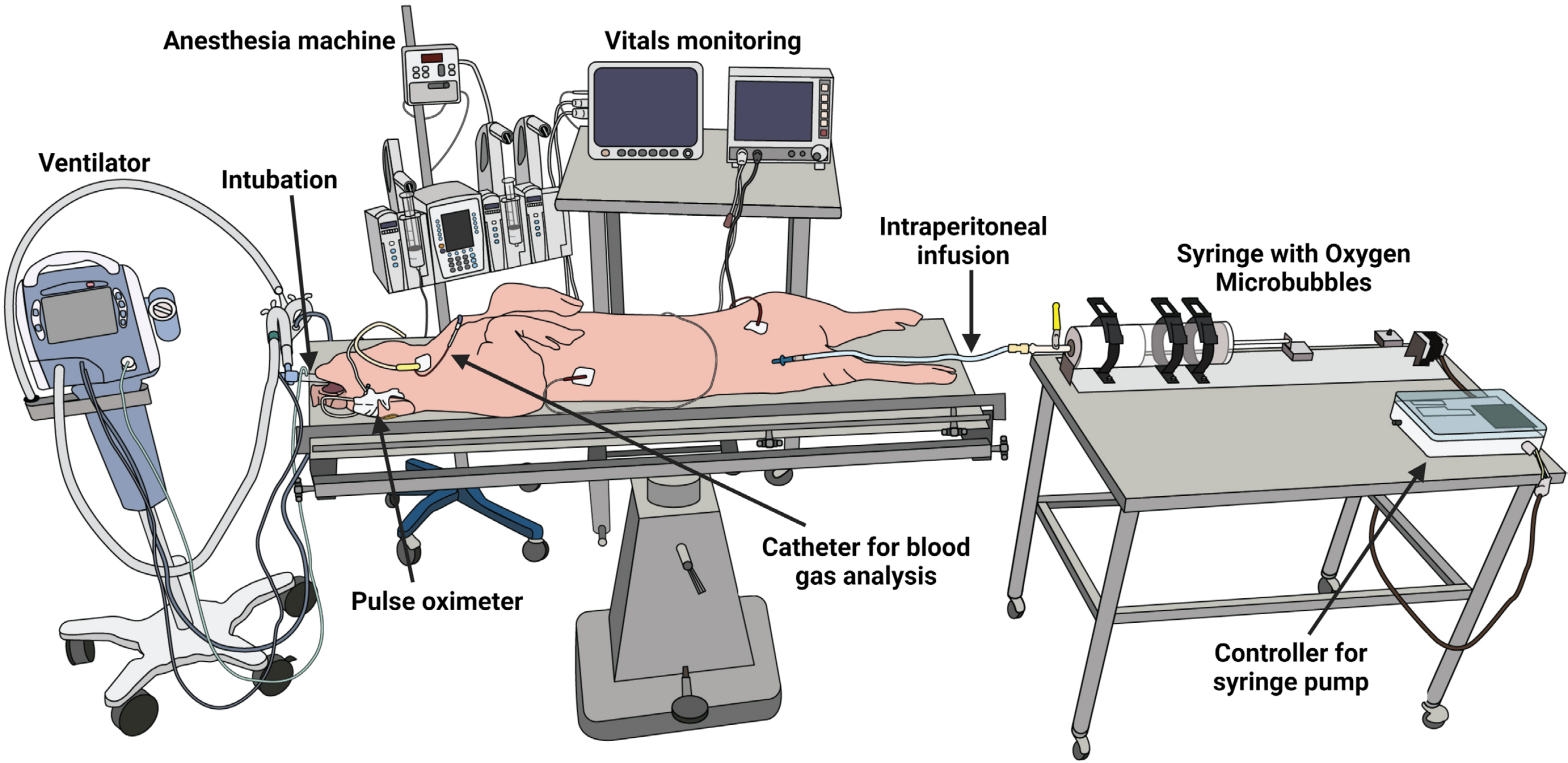
Schematic diagram depicting the porcine intensive care unit setup

Before conducting the experiments, a fully functioning ICU was established to develop surgical intensive care capabilities for maintaining a large pig ARDS injury model in a critical care scenario. The ICU capabilities were designed to test the OMB therapy to mimic the clinical respiratory failure scenario. The porcine ICU consisted of a large animal anesthesia and resuscitation ventilator, vitals monitor, and relevant accessories. A schematic of the porcine ICU setup is shown in Figure 2.

### 2.3 Presurgical preparation

Once a fully functional ICU was set up, the pigs were prepared for the study according to the groups listed above. First, all the pigs were fasted 8 h before starting the study to prevent asphyxiation from emesis during intubation. At the start of the study, the pigs were weighed and then sedated with an intramuscular injection (IM) of 0.05 mg/kg Telazol/Ketamine/Xylazine (TKX; Zoetis; 501072, VetOne; 510004, VetOne). Next, IM atropine (0.05 mg/kg; 510221, VetOne) was given to reduce salivation during intubation. Following sedation, the animals were placed on a surgery table and intubated with an endotracheal tube (6.5–7.5 mm, VetOne). The pigs were placed in the supine position on a warming pad (AP‐AV‐057 and TP‐700, Stryker) set at 38°C to maintain the pig's body temperature. Next, benzocaine (61095, Cetylite) was sprayed into the trachea to reduce gag reflexes from intubation. Subcutaneous injection of lidocaine (MWI Animal Health; 83705) was then administered at the surgery site. Finally, a urinary catheter (37432, Jorgensen) was placed in the urinary bladder to allow for drainage (≥1 ml/kg/h). In the event of distress caused by pain, 3 ml of benzocaine or lidocaine were administered under the surgeon's discretion.

### 2.4 Surgical catheter placement for monitoring vitals

Following the presurgical preparation, the left and right sides of the neck and the right leg were shaved and sterilized for surgery. A triple‐lumen catheter (1720, MILA International, Inc.) was placed in the left internal jugular vein. After the catheter was placed, propofol (46053, Zoetis) was infused at a constant rate of 0.2–0.4 mg/kg/min via the first lumen. The second lumen was then connected to a 0.4–0.7 mg/kg/min supply of midazolam (47786, Akorn) mixed with a 0.03–0.10 mg/kg/min supply of fentanyl (45741, West‐Ward). A syringe pump (Alaris 8015 PCU and Alaris 8110 Syringe Module, CareFusion) was used to regulate the continuous infusion of all anesthetic drugs. To maintain proper fluid levels, the third lumen was connected to physiological saline (150 ml/h, 510224, VetOne). The endotracheal tube was then attached to a mechanical ventilator (Newport HT70 Plus Ventilator, Medtronic).

A second triple‐lumen catheter (1720, MILA international) was placed in the right carotid artery for carotid blood sampling and arterial pressure monitoring. Then, an introducer sheath (AK‐09903‐CDC, Teleflex) was placed in the right jugular vein through the same incision site. Using the introducer sheath, a Swan‐Ganz catheter (746F8, Edwards Lifesciences) was positioned in the pulmonary artery (PA) and connected to a hemodynamic monitor (Vigilance II, Edwards Life Sciences) for mixed venous blood sampling, PA pressure monitoring, and for measuring the cardiac output, central temperature, and mixed venous oxygen saturation (SvO_2_). A single lumen catheter (SA1806, MILA International) was then percutaneously placed in the right femoral artery using an ultrasound machine (Site Rite IV, Bard) for femoral blood sampling.

### 2.5 Vitals monitoring

After the surgical placements were completed, medical devices were connected to monitor the vitals. A veterinary monitor (V9203, Smiths Medical) was used to display the vitals via six sensors: (1) a pulse oximetry sensor (V1700, Smiths Medical) was placed on the tail to measure the peripheral oxygen saturation (SpO_2_), (2) an air gas collection tube (WW1100, Smiths Medical) was connected to the endotracheal tube to measure the end‐tidal CO_2_ (EtCO_2_), (3) a thermometer (WWV3418, Smiths Medical) was placed rectally to measure the internal body temperature, (4) a three‐lead electrocardiogram cable (V3110TLS, Smiths Medical) was placed to measure the heart rate and to obtain an ECG waveform, (5) an invasive blood pressure transducer (V6402, Smiths Medical) was connected to the triple‐lumen catheter in the carotid artery to measure the mean arterial pressure, and (6) a second transducer (V6402, Smiths Medical) was connected to the Swan‐Ganz catheter to measure the pulmonary artery pressure. In addition, raw data from the veterinary monitor were collected every 30 s.

Following the surgery for monitoring vitals and subsequent ventilation, a catheter for OMB infusion was placed in either the thoracic cavity (IT, CTT28, Mila International, Inc.) or the peritoneal cavity of the abdomen (IA, CTT28, Mila International, Inc.) according to the experimental group. The IT and IA catheters were placed using the trocar method. While placing these catheters, proper pressures were maintained utilizing a pressure transducer (CTC‐6F, Gaeltec Devices Ltd.). In addition to the infusion catheters, a pigtail catheter (PIG1260T, CareFusion) was attached to a chest drain (Oasis Dry Suction Chest Drains, Atrium Medical) to prevent pneumothorax. For the IT placement of the catheter, a pigtail drainage catheter (PIG1260T, CareFusion) was placed in the right cervical pleura using the Seldinger technique, followed by positioning a chest tube in the costodiaphragmatic recess of the pleural cavity. Similarly, a chest tube was placed in the right lower quadrant of the peritoneal cavity for the IA catheter placement.

### 2.6 Mechanical ventilation

The ventilator was then operated under the volume control mode (tidal volume 8 ml/kg, respiratory rate 25, 5 cm H_2_O PEEP) and set to 100% FiO_2_ during surgical procedures. Following the surgery, pigs were weaned to room air‐equivalent oxygen supplementation by reducing the fraction of inspired oxygen (FiO_2_) on the ventilator by decrements of 10%, until a FiO_2_ of 21% was reached. About 3 min of acclimation was allowed between the adjustments. Throughout the remainder of the experiment, the ventilator settings were titrated to the pigs' response to maintain eucarbic (normal blood CO_2_) conditions. If the EtCO_2_ increased beyond 60 mmHg, the tidal volume was increased to bring the EtCO_2_ back to normal (35–45 mmHg) levels. In addition, the pigs were rotated every 2 h after nebulization to reduce compression of the lungs during the study.

### 2.7 Blood sampling for arterial blood gas (ABG) analysis

After oxygen weaning, baseline blood samples were taken every 10 min from all arterial catheters until a ±5% stability in the PaO_2_ was recorded. Blood samples were then collected from the same catheters every 30 min for the remainder of the study. For the ABG analysis, 0.5 ml of arterial blood was collected and analyzed for oxygen partial pressure (PaO_2_), oxygenated hemoglobin concentration (O_2_Hb), and blood oxygen saturation (SaO_2_), using a co‐oximeter (ABL80‐FLEX CO‐OX Analyzer, Radiometer). After collecting a blood sample, 1 ml of physiological saline (510224, VetOne) and 0.5 ml of heparinized saline (100 IU/ml; Heparin Sodium, Sagent) was flushed through the catheter to prevent clotting. The PaO_2_ values were averaged over the entire study duration and plotted.

### 2.8 Preparation and administration of LPS

After oxygen weaning, an LPS solution was prepared by dissolving powdered LPS (*Escherichia coli* 0111: B4, L2630, Sigma‐Aldrich) in physiological saline (510224, VetOne) to obtain a final concentration of 20 mg/ml. A stirring hot plate (Corning Inc.) set to 90°C was used to homogenize the mixture at 200 RPM and heat it to a final temperature of 45°C. The LPS solution was then nebulized into the administration circuit and into the animal over a 2 h period after the baseline blood samples were obtained. The nebulizer in the circuit (Air, Airgas) was connected to an outlet with a gas flow rate of 3 L/min, in line with the mechanical ventilation breathing circuit. All the ARDS group and subgroup pigs (ARDS‐only, ITAS, IAAS, ITAO, IAAO) were administered LPS intratracheally at a 4 mg/kg dosage via the jet nebulizer (UniHEART Universal Nebulizer, Westmed Inc.). Following the administration of LPS, the pigs were monitored for the symptoms of moderate ARDS, that is, a PaO_2_/FiO_2_ ratio <200 mmHg (Berlin criteria) or a SpO_2_ below 80% persistent for 30 min within 4 h.

### 2.9 Synthesis of OMBs

The lipid OMBs were synthesized as explained by Feshitan et al. (Feshitan et al., 2014). Briefly, phospholipid 1,2‐distearoyl‐sn‐glycero‐3‐phosphocholine (DSPC; NOF) and polyoxyethylene‐40 stearate (PEG‐40S; Sigma‐Aldrich) were combined in a 9:1 molar ratio. The mixture was then dissolved in 0.2 um PBS solution (0.15 M) to obtain a final concentration of 10 mg/ml. Next, the mixture was heated to 65°C and dispersed in a sonifier (Danbury). The resulting solution was then refrigerated until further use. The above lipid suspension was combined with oxygen by feeding it to an ultrasonic horn reactor enclosed in a water cooler. The oxygen was emulsified at full sonication and then fed to a floatation column to separate the resulting OMBs at the bottom and macrofoam at the top. The OMBs were isolated in syringes by centrifuging them at 130 rcf for 30 min.

### 2.10 Administration of saline and OMB

After developing the symptoms of ARDS, the pigs received infusions of OMB or saline. The OMB was synthesized as described previously (Swanson & Borden, 2010). All infusions were made following the experimental timeline. Using a lab‐built syringe, saline and OMB infusions were initiated 15 min after meeting the moderate ARDS criteria. Pigs in the ITAO group were given 500 ml of OMB via the IT catheter, whereas pigs in the IAAO group were given a 100 ml/kg bolus of OMB via the IA catheter. Similarly, pigs in the ITAS and IAAS groups received the same volume of saline via IT and IA catheters, respectively. All the infusions were made with the syringe pump at a flow rate of 500 ml/min.

### 2.11 Euthanization of pigs

The study endpoint corresponded to a time point of 8 h after the oxygen was reduced to 21% or an event of cardiac arrest. At 8 h postoxygen reduction, all the surviving pigs were humanely euthanized with a lethal injection of pentobarbital sodium (15199, Vortech).

### 2.12 Chest radiography

Chest radiographs of lungs were taken before the surgeries and postmortem with the pigs in the ventrodorsal and left lateral positions to evaluate for the pathognomonic signs of ARDS. The veterinary staff performed the x‐ray procedure using a mobile x‐ray machine (EPX‐2800, Ecotron). The x‐ray images were then examined and scored for the presence of pulmonary edema, alveolar consolidation, and bilateral infiltrates in the lungs. The procedure developed by Warren et al. was used for scoring the radiographs (Legband et al., 2014).

### 2.13 Lung histological studies and scoring

Following the death or euthanization of the pigs, a necropsy was performed to collect samples of the trachea and lungs. The first set of 1 cm × 5 cm sections was excised from each lung lobe to obtain the samples. These samples were then immediately fixed in 10% neutral buffered formalin (575A‐1GL, Medical Chemical). Following fixation, a cross‐section from each lobe was collected and processed on a Tissue‐Tek VIP 5 Tissue Processor (Sakura Finetek USA, Inc.). The tissues were then embedded in paraffin blocks and sectioned 4 μm thick on an HM 355S Thermo Scientific Microtome (Thermo Scientific). The slides were routinely de‐paraffinized and stained using hematoxylin and eosin (Leica ST5020 H&E, Leica Biosystems Inc.), following which glass coverslips were placed on the samples. A board‐certified veterinary anatomic pathologist performed all histological evaluations to assign lung injury scores. The scores were then averaged over 10 random 400X fields of view. All histological evaluations were blinded.

Each lung section was scored for the presence of hemorrhage, edema, and inflammation, then assigned a score of 0–3 depending on the severity using the method reported by Erdem et al. (Kohman et al., 2010). A score of 0 represented normal tissue. A score of 1 represented mild pathological changes characterized by edema or hemorrhage within 5%–10% of the lung or an influx of fewer than 10 leukocytes per 400X field of view. As the grading score does not differentiate between the types of leukocytic infiltrates, some lobes assigned a score of 1 contained a few exfoliated macrophages. A score of 2 indicated moderate pathological changes with edema or hemorrhage affecting 10%–20% of the lung or 10–45 leukocytes per field of view. A score of 3 represented severe pathological changes with edema or hemorrhage affecting >20% of the lung or an influx of more than 45 leukocytes per field of view. The second set of tissue samples was then excised from each lung lobe and weighed, followed by placing them in an incubator (20GC Analog Lab Oven, Quincy Lab) maintained at 75°C. The samples were weighed daily. When the weights had stabilized, the dry weight was recorded. The wet/dry ratio was then calculated.

### 2.14 Statistical analysis

The subgroups in control and ARDS‐only groups were compared and combined into a single “control” and “ARDS‐only” group when differences in the vitals were not observed. For %SpO_2_, PaO_2,_ histology, and chest radiography results, data from all six study groups (Control, ARDS‐only, ITAS, IAAS, ITAO, and IAAO) were first analyzed to assess the normality using Q‐Q plots. Following the normality test, a one‐way ANOVA was performed for data with standard deviations (as shown by the Brown‐Forsythe test) that did not significantly differ among the groups. When the standard deviation of the data was significantly different among the groups, the Brown‐Forsythe and Welch ANOVA test was used for analysis. Next, Tukey's multiple comparisons test was used to compare the means of individual groups. Finally, a Kaplan–Meier plot was used to study the survival of the pigs. Statistical tests were performed on the survival analysis using the log‐rank test with Bonferroni's correction. An alpha value <0.05 was considered statistically significant. The data plotting was performed using MATLAB (MathWorks) and GraphPad Prism (GraphPad Software, Inc), while statistical testing was performed using GraphPad Prism.

## 3 RESULTS

### 3.1 Development of a rapid‐onset moderate‐to‐severe ARDS injury model in a porcine ICU

A surgical ICU was successfully set up to develop protocols and capabilities for maintaining a large pig model of rapid‐onset, moderate‐to‐severe ARDS. The ARDS definition from the Berlin criteria requires a PaO_2_/FiO_2_ ratio of below 300 mmHg for mild ARDS, below 200 mmHg for moderate ARDS, and below 100 mmHg for severe ARDS. For the pigs in the ARDS‐only group, the PaO_2_/FiO_2_ ratio dropped below 200 mmHg in 3.53 ± 1.45 h, indicating that the pigs developed clinically relevant moderate ARDS. The PaO_2_/FiO_2_ ratio further dropped to 160 ± 24 mmHg in the next 2 h. Additionally, the mortality rate of the pigs after 4.08 ± 0.68 h was 12.5%.

**FIGURE 3:**
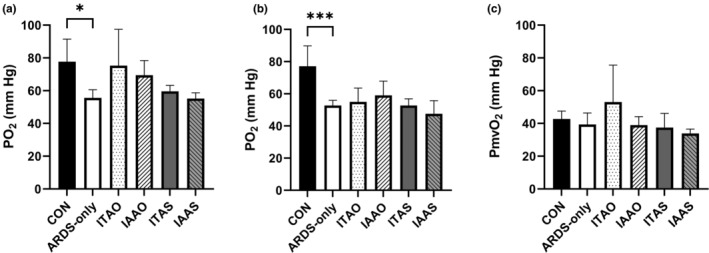
PaO_2_ values of pigs in different groups. (a) The femoral artery PaO_2_ level was significantly greater in the control group than in the ARDS‐only group. The difference in PaO_2_ levels after oxygenated microbubble administration was not statistically significant (*p < 0.05). (b) The carotid artery PaO_2_ values were significantly greater in the control group compared with the lipopolysaccharide group (***p < 0.001). (c) The PmvO_2_ levels in the pulmonary artery did not show any significant difference between the groups (*p* > 0.05).

**FIGURE 4:**
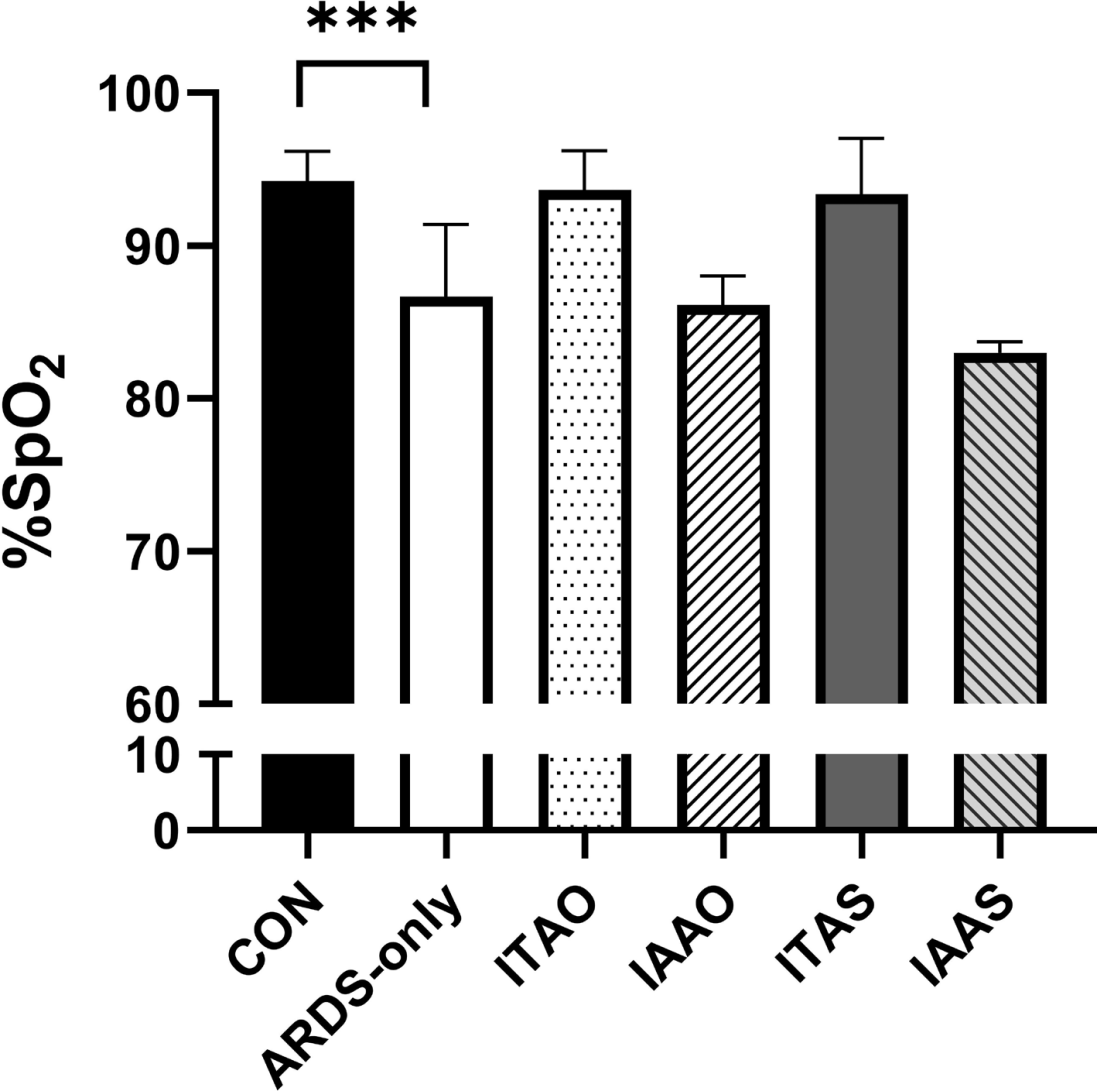
Pulse oximetry values. The % SpO_2_ values dropped starting at 2 h after lipopolysaccharide administration until the end of the experiment. The difference was statistically significant (***p < 0.001).

The peripheral oxygen saturation (SpO_2_) was measured as a non‐invasive reading of blood oxygenation. In this study, blood oxygenation continuously decreased for all the groups starting at 0.5 h post‐LPS administration. Once the FiO_2_ was titrated down to 21%, the PaO_2_ levels of all LPS‐treated groups (ARDS‐only, ITAS, IAAS, ITAO, and IAAO) dropped to 70 mmHg by 2 h, indicating moderate ARDS. Figure 3 shows the PaO_2_ values of the pigs averaged over the entire study duration. The PaO_2_ in the femoral artery was significantly lower in the ARDS‐only group than in the control group (*p = 0.016; Figure 3a). Similarly, the PaO_2_ in the carotid artery of the ARDS‐only group was markedly lower than that of the control group (***p = 0.0007; Figure 3b). For the samples matched to the carotid artery, %SpO_2_ values indicated a significantly lower oxygen saturation in the ARDS‐only group than in the controls (***p = 0.001; Figure 4).

### 3.2 Variation in heart rate from infusions

**FIGURE 5:**
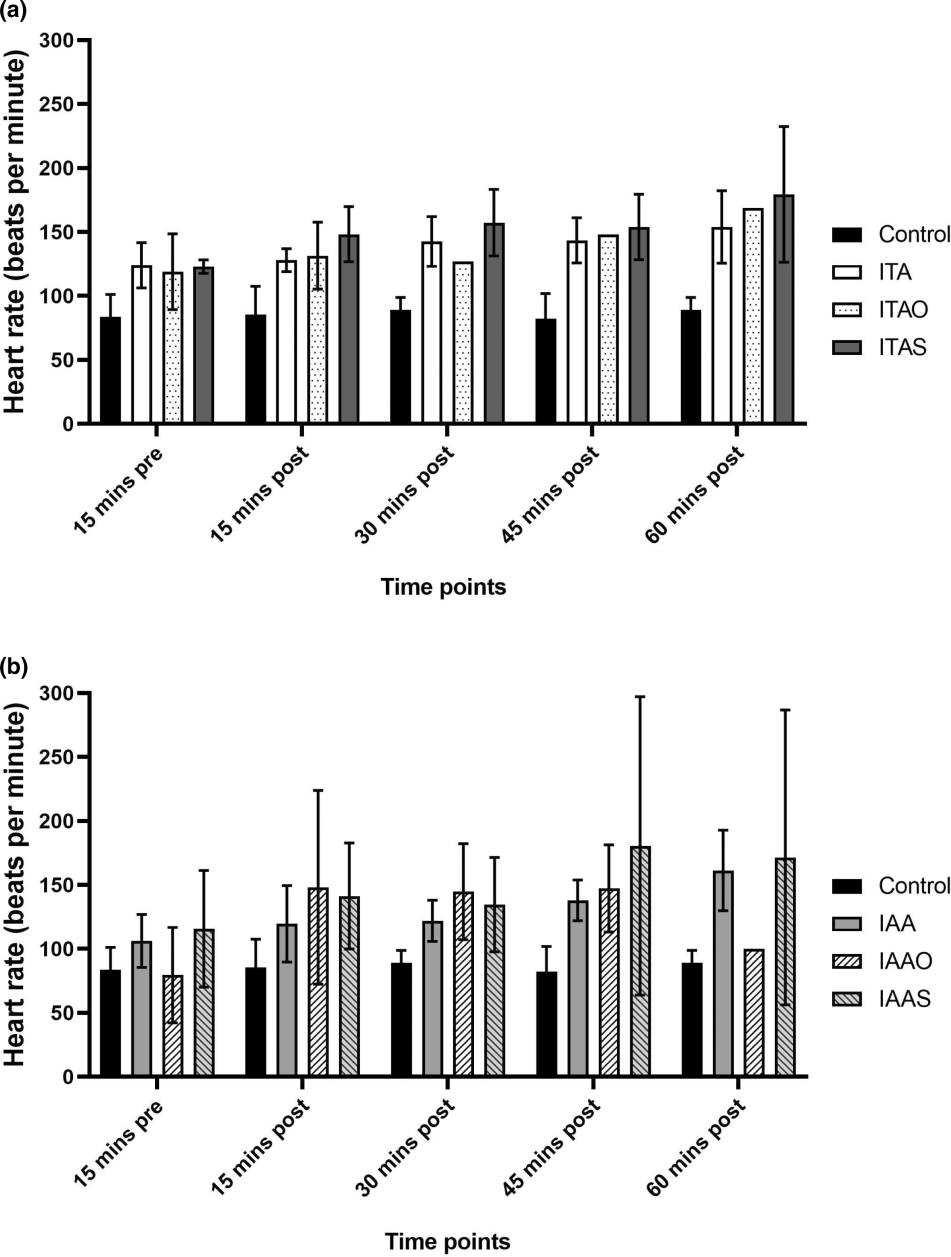
Heart rates pre‐ and postinfusions in subgroups of (a) intrathoracic catheter and (b) intraperitoneal administration catheter

The heart rates of all the pigs were continuously monitored from the start of the experiment. Heart rate values 15 min before the OMB infusions were compared to those at 15, 30, 45, and 60 min after the infusion of OMB or saline. The mean heart rate of the control pigs was the lowest (Figure 5a,b), with values continuously below 100 beats per minute without signs of tachycardia (heart rate >100 beats per minute). However, the other subgroups (IT and IA) experienced tachycardia. Further, since the difference in the heart rate between ARDS‐only groups (ITA, IAA) and infusion groups (ITAO, IAAO, ITAS, IAAS) was not significant, the increased heart rates compared with the control group can be attributed to the ARDS model.

### 3.3 Peripheral blood oxygenation and partial pressure of oxygen post‐OMB infusions

OMB was administered 15 min after the Berlin criteria were met. Since the FiO_2_ was 21% after the study, the Berlin criteria are then essentially the shifted PaO_2_ values (PaO_2_/0.21). Post‐OMB administration, the femoral artery PaO_2_ values for the ITAO and IAAO groups did not differ significantly (*p* = 0.984; Figure 3a). Also, the ITAO and IAAO PaO_2_ levels were not significantly different from the ITAS (*p* = 0.563) and IAAS (*p* = 0.591) groups, respectively. In addition, the PmvO_2_ and PaO_2_ values in the pulmonary and carotid arteries, respectively, showed similar results (Figure 3a–c). Moreover, the PaO_2_ values of the ARDS‐only group were not significantly different from the treatment groups (ITAO, IAAO, ITAS, IAAS). Similarly, %SpO_2_ values in the ITAO and IAAO groups were not significantly different from the ITAS (*p* > 0.999) and IAAS groups (*p* = 0.738), respectively. However, the %SpO_2_ of pigs in the ITAO group was greater than the IAAO group (*p = 0.032; Figure 4). Table 1 shows a comparison of the PaO_2_ and SpO_2_ values for the ARDS‐only and OMB infusion groups. Taken together, the above results show that the OMB infusion, irrespective of the route, did not supplement the oxygenation levels in this rapid‐onset moderate‐to‐severe ARDS pig model.

**TABLE 1:** Comparison of the (a) PaO_2_ and (b) SpO_2_ values in the ARDS‐only and OMB infusions groups

PaO_2_ (mmHg)	ARDS‐only (1)	ITAO (2)	IAAO (3)	*p* value
(a)				
Femoral	55.6 ± 4.9	75.3 ± 22.2	69.5 ± 8.9	1 versus 2: 0.19 1 versus 3: 0.45
Carotid	52.7 ± 3.3	54.9 ± 8.6	59.1 ± 8.8	1 versus 2: 0.99 1 versus 3: 0.89
Pulmonary	39.4 ± 6.9	53.0 ± 22.6	38.9 ± 5.1	1 versus 2: 0.97 1 versus 3: 0.99
(b)				
%SpO_2_	86.7 ± 4.7	93.6 ± 2.6	86.1 ± 1.9	1 versus 2: 0.03 1 versus 3: 0.99

Abbreviation: ARDS, acute respiratory distress syndrome.

### 3.4 Kaplan–Meier survival analysis

**FIGURE 6:**
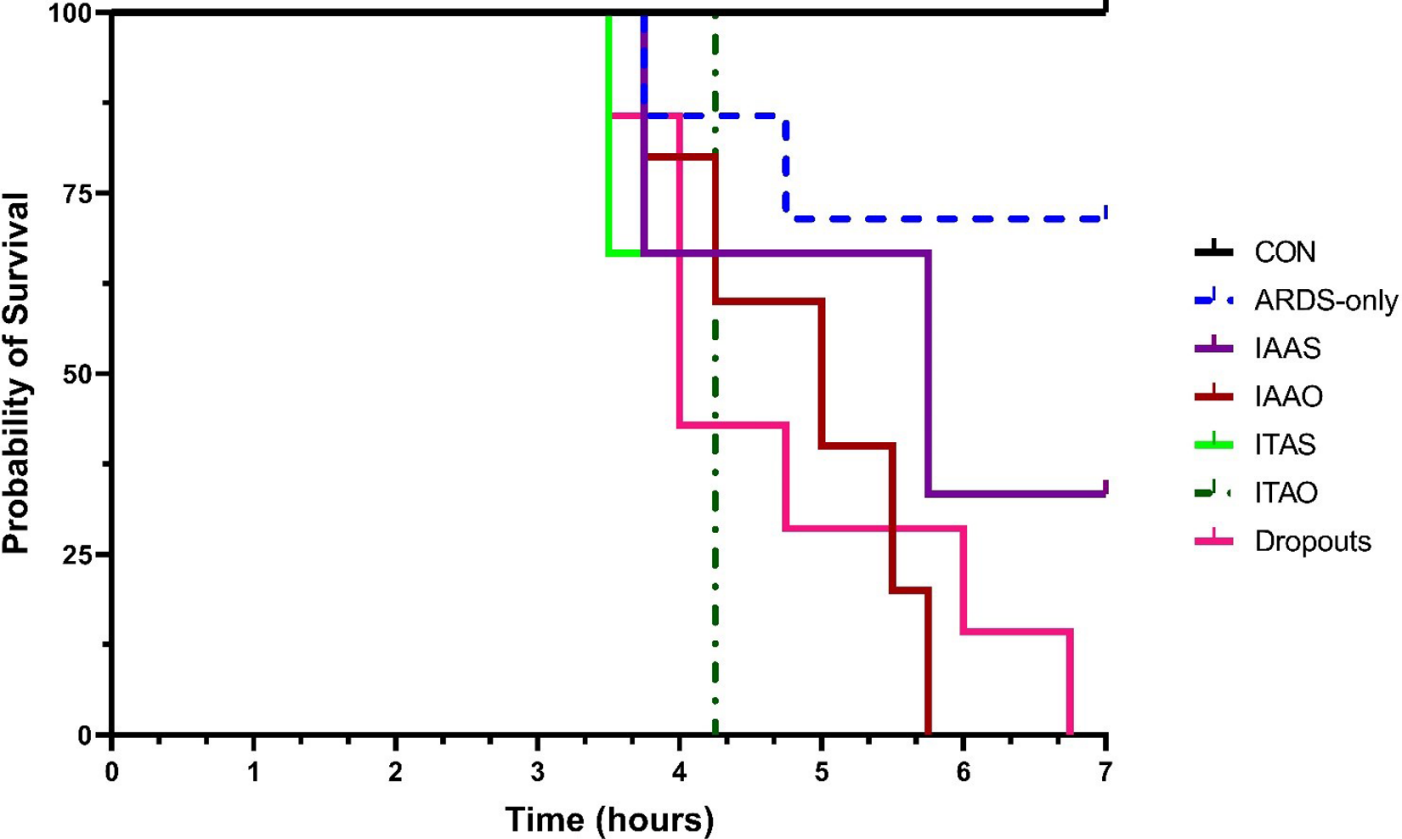
Kaplan–Meier survival plot. Pigs in the control group showed a 100% survival rate after 7 h from the start of the study. Pigs in the ITAO group had a lower survival rate than their acute respiratory distress syndrome (ARDS)‐only and ITAS counterparts. However, the difference did not reach significance (*p* > 0.05). Pigs in the IAAO group had a lower survival rate than the pigs in the ARDS‐only group (*p < 0.05). The IAAS and ITAS groups showed similar survival rates after 3.5 h as seen from the overlapping curves. The differences between other groups were not significant.

A Kaplan–Meier plot was generated based on the survival of pigs at different time points post‐LPS administration. The “dropouts” refer to the pigs that were excluded from the study for various reasons, for example, failed catheters, abnormal anatomy, and surgery complications. Pigs in the control group showed 100% survival at 7 h post‐LPS. With the FiO_2_ reduced from 100% to 21%, ARDS‐only pigs experienced a mortality of >25% after 7 h. Surprisingly, pigs in the IAAO group had a survival rate of 0% at 4.2 h post‐LPS, significantly lower than the survival rate of pigs from the ARDS‐only group (*p < 0.05). Similarly, the ITAO group pigs had a survival rate of 0% at 5.7 h post‐LPS, which was lower than the survival rate of the ARDS‐only group. However, this difference did not reach statistical significance. The survival rates of pigs in the ITAS and IAAS groups did not show any statistically significant differences compared with the ARDS‐only group. Additionally, the ITAS and IAAS groups showed a similar survival rate beyond 3.75 h, as seen from the overlapping survival curves (Figure 6).

### 3.5 Chest radiography scores

**FIGURE 7:**
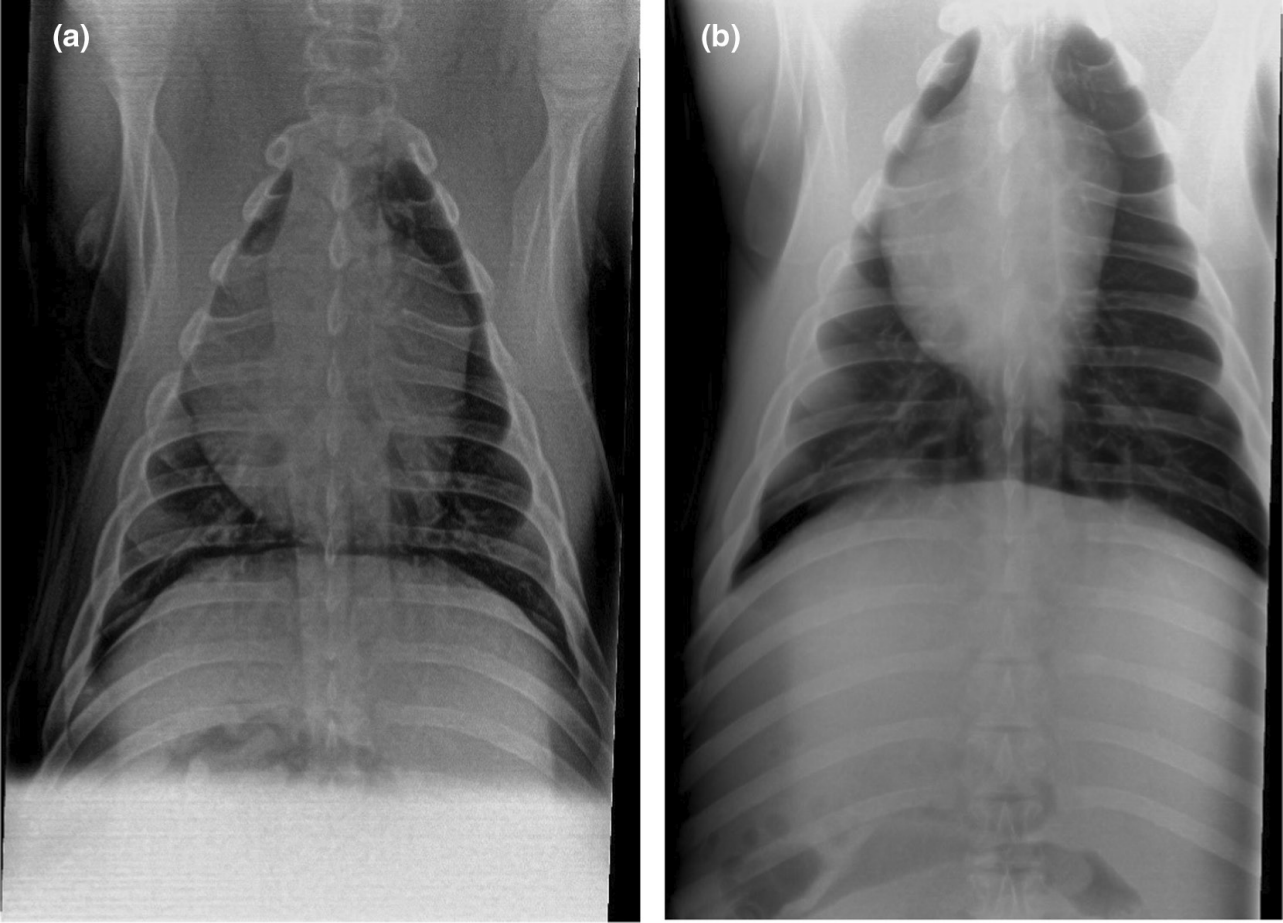
Representative radiograph images (supine position) showing no significant signs of pulmonary edema. (a) Prestudy x‐ray of a control animal and (b) Postmortem x‐ray of the same control animal

**FIGURE 8:**
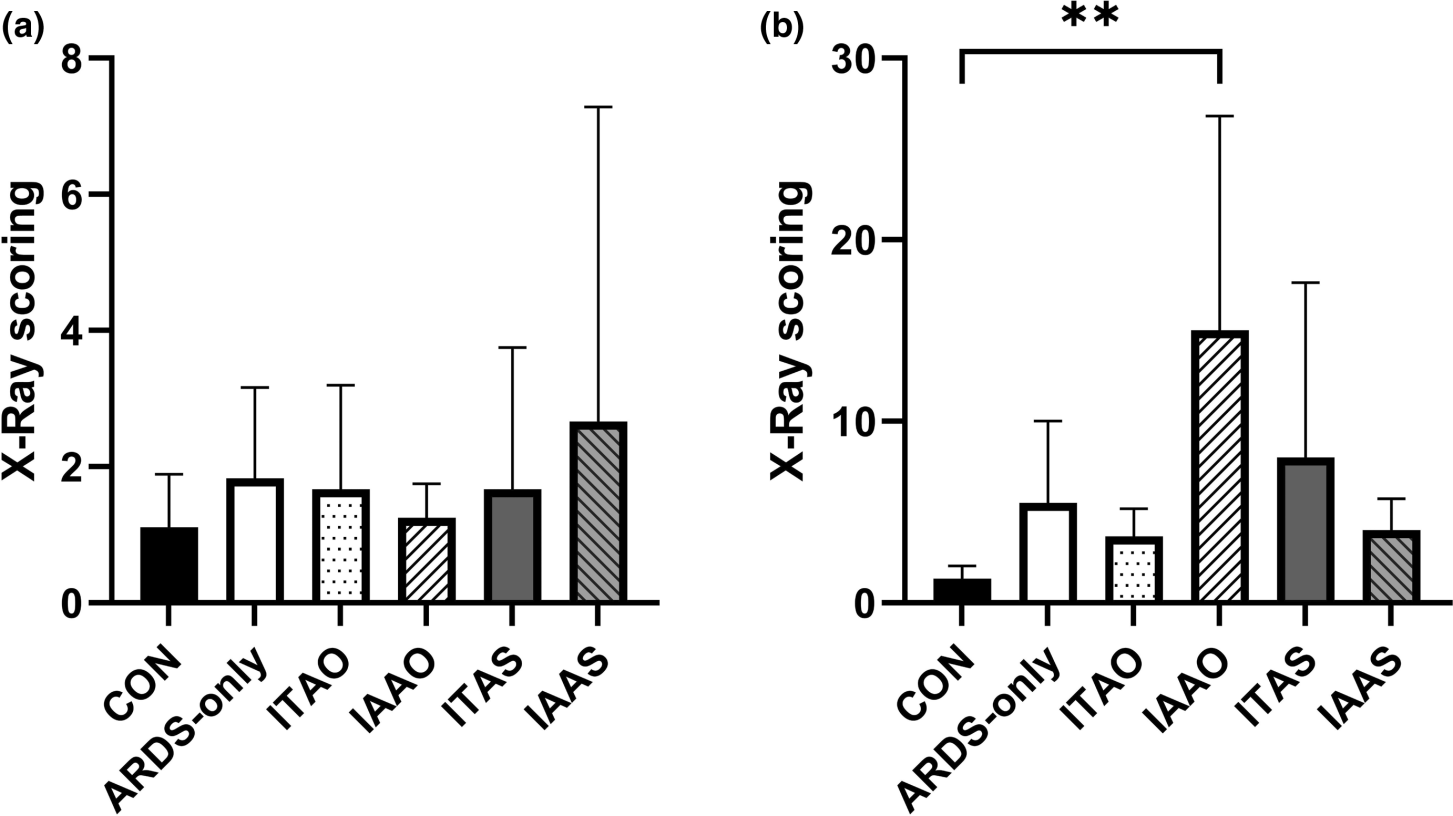
Prestudy and poststudy radiograph scores. (a) The prestudy radiograph scores did not show a statistical difference between the groups. (b) The poststudy radiograph score was greater in the lipopolysaccharide group but the difference compared with controls was not statistically significant (*p* > 0.05). The radiograph score in the oxygenated microbubble group (IAAO) was significantly greater than controls (**p < 0.01)

To further investigate the onset of ARDS and accumulation of the LPS irritant in the lungs, chest radiographs of pigs were taken and scored (Figure 7a,b). Pig radiographs from the control and ARDS‐only groups had a similar prestudy score of 1.1 ± 0.8 and 1.8 ± 1.3, respectively (*p* = 0.16). The poststudy radiograph scores for the same groups were 1.3 ± 0.7 and 5.5 ± 4.5, respectively (Figure 8a,b). Although the poststudy score of the ARDS‐only group was greater than controls, the difference was not statistically significant (*p* = 0.740). The IAAO group radiography score poststudy was the only group significantly greater than the control group (**p = 0.007).

### 3.6 Histology scores and wet/dry ratio for the lungs

**FIGURE 9:**
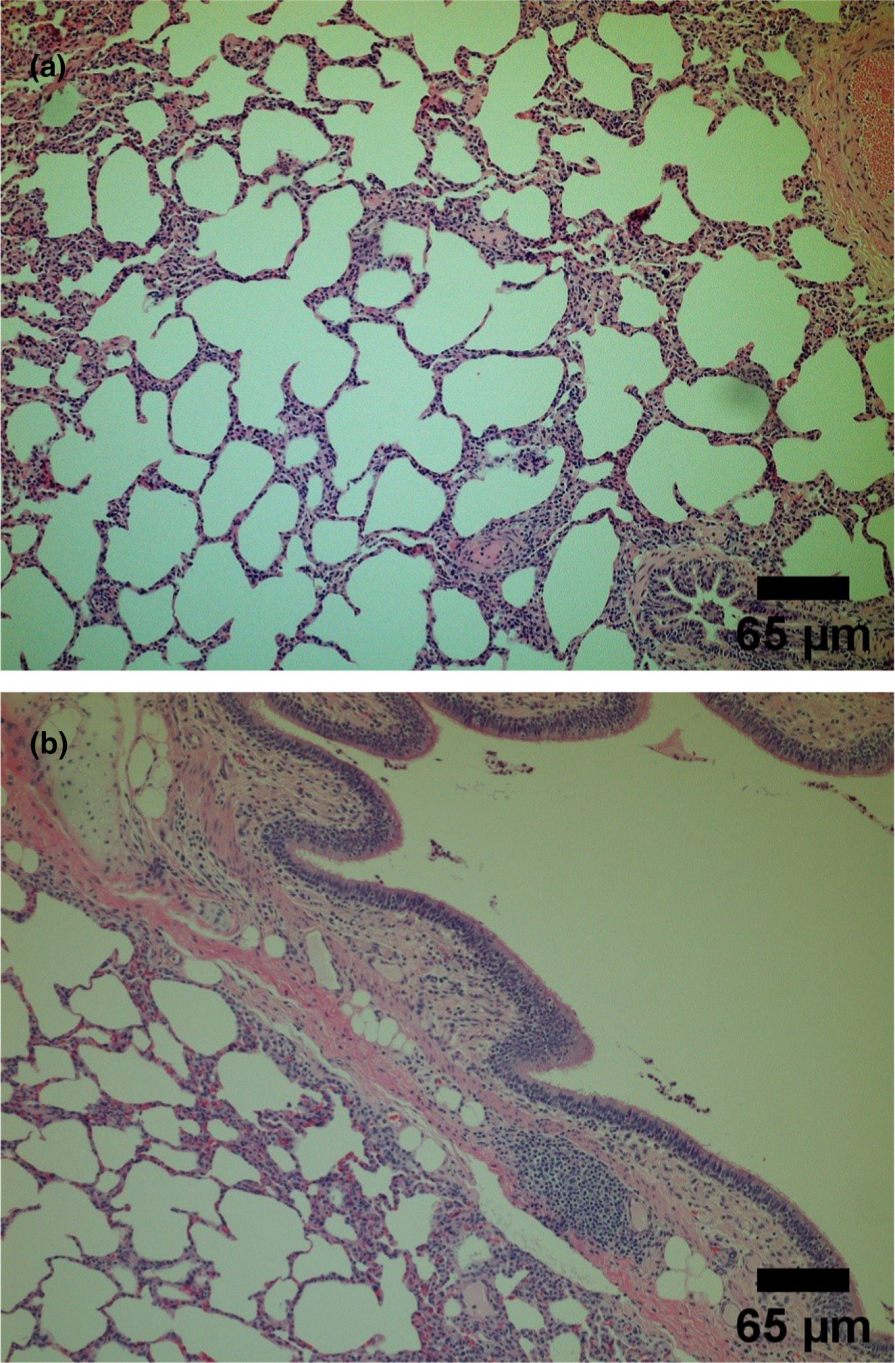
Representative histological images for the control group with no statistically significant difference (each scale bar measures 65 μm). (a) Image with a score of 0 for inflammation and (b) Image with a score of 1 for inflammation

**FIGURE 10:**
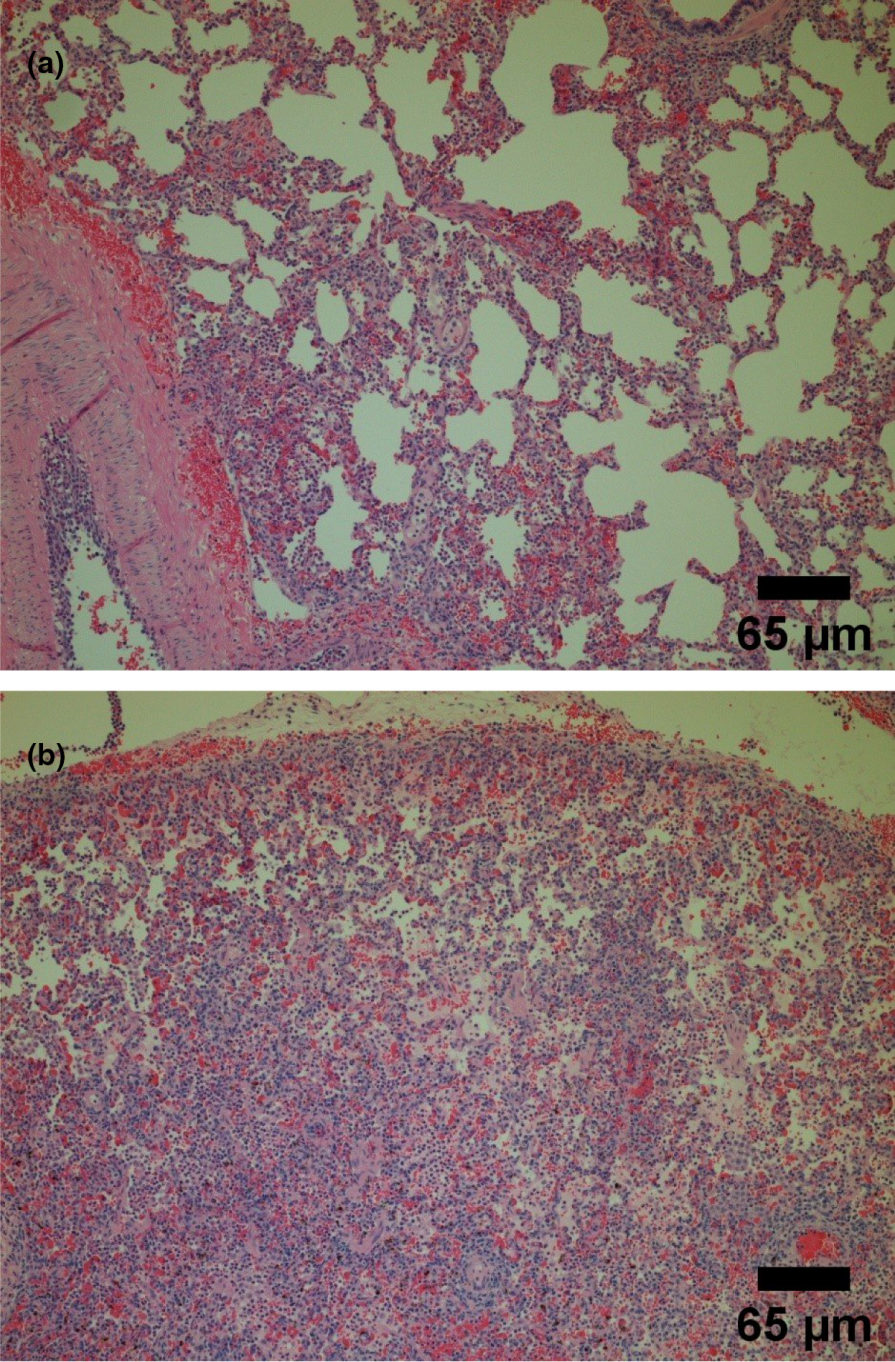
Representative histological images for the lipopolysaccharide group with a significantly greater incidence of inflammation (each scale bar measures 65 μm). (a) Image with a score of 2 for inflammation and (b) Image with a score of 3 for inflammation

**FIGURE 11:**
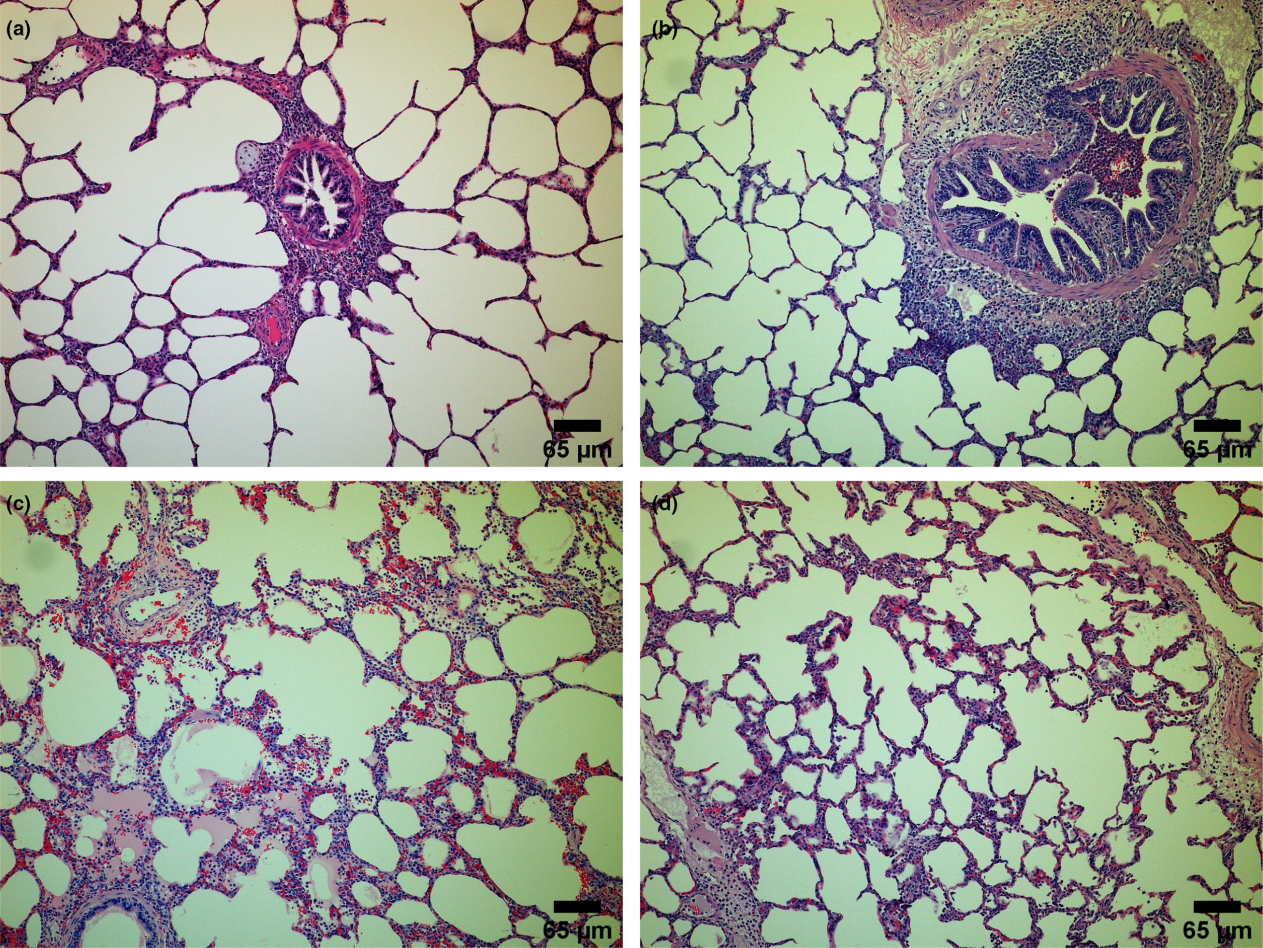
Representative histological images for treatment groups (each scale bar measures 65 μm). (a) Image with a score of 1 for inflammation from ITAO group, (b) image with a score of 2 for inflammation from IAAO group, (c) image with a score of 1 for inflammation from ITAS group, and (d) image with a score of 2 for inflammation from IAAS group

**FIGURE 12:**
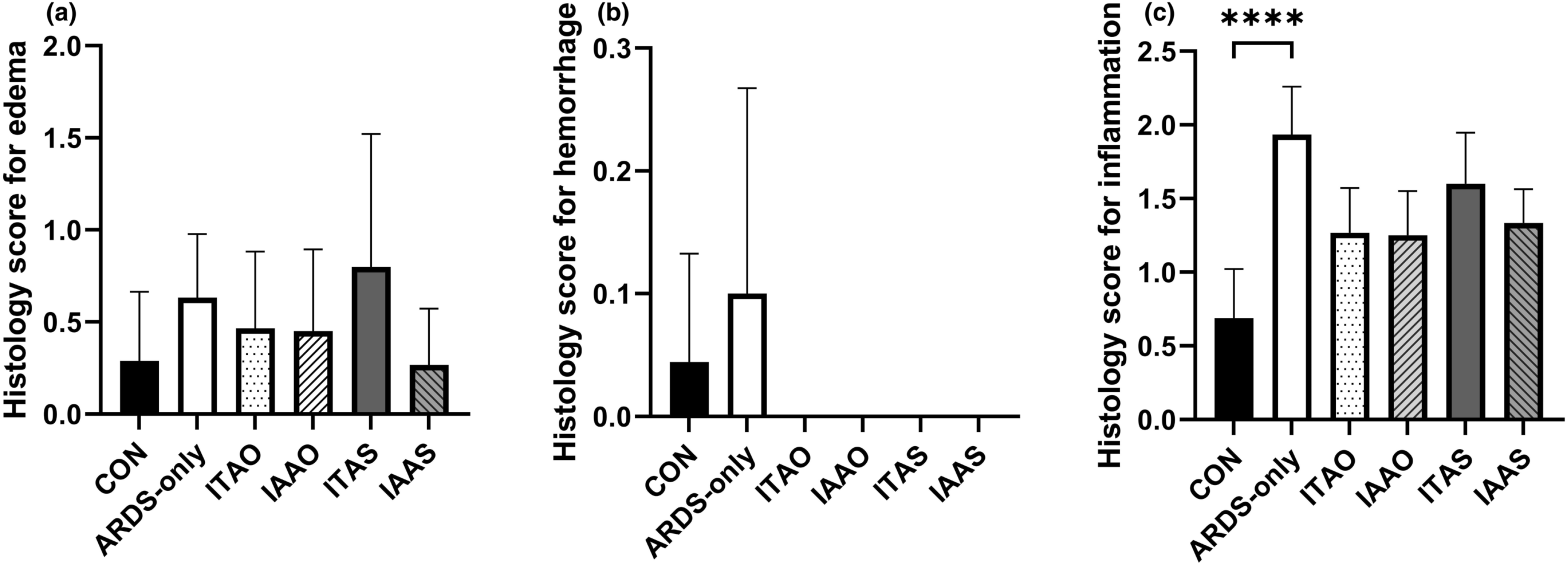
Histology scores for edema, hemorrhage, and inflammation. (a) The histological score for edema was not significantly different among the groups, (b) the histological score for hemorrhage was not significantly different among the groups, and (c) the histological score for inflammation was significantly greater in the lipopolysaccharide group compared with controls (***p < 0.001).

**FIGURE 13:**
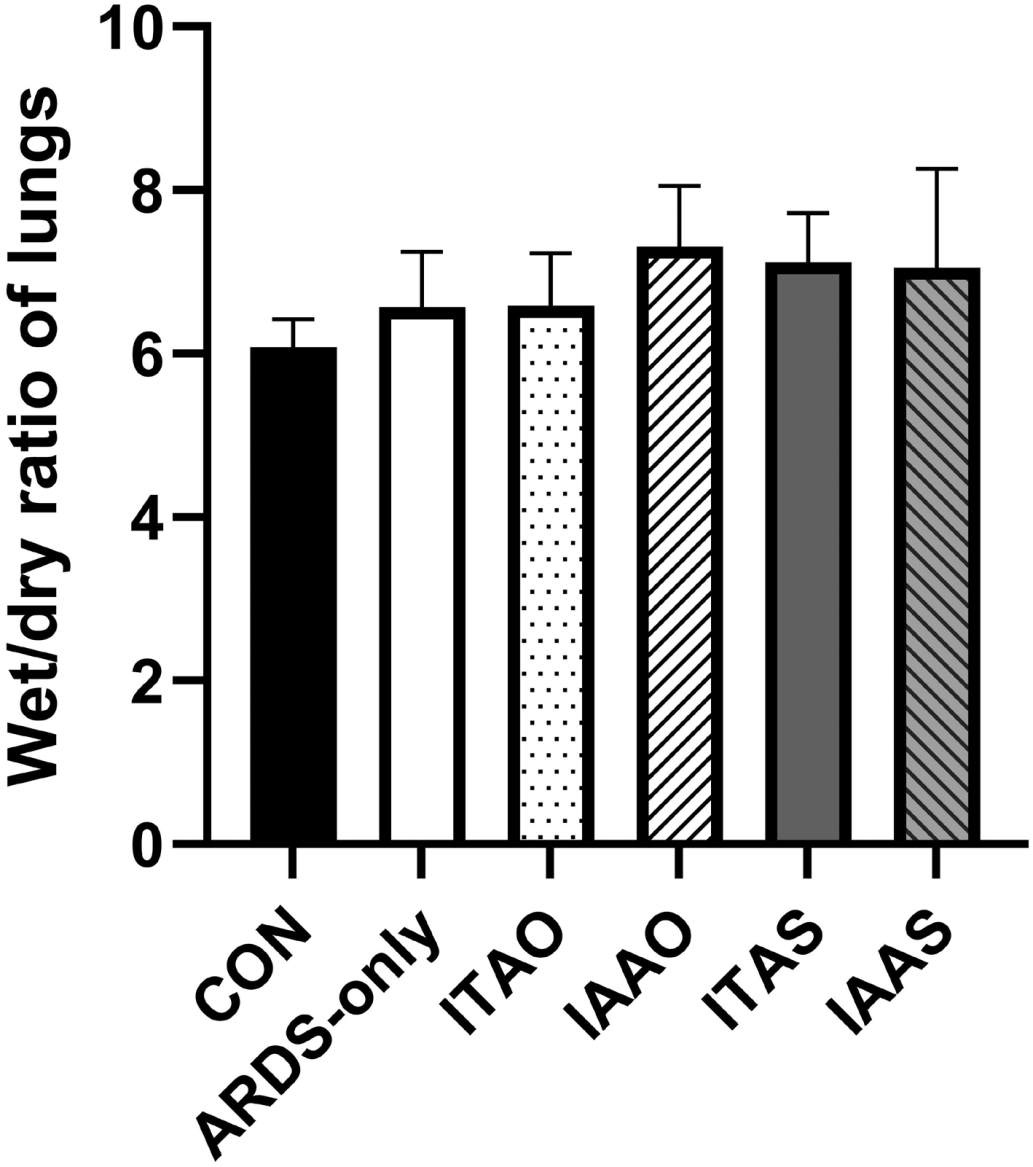
Wet/dry lung ratios of pigs postmortem

Posteuthanization of pigs, the histological scores, and wet/dry ratios of the lungs were calculated. A histology score represents the number of leukocytes per field of view. Figures 9, 10, and 11 show representative histological images. The scale bar for each image measures 65 μm. Figure 10a,b shows an image with a score of 0 and 1 for inflammation, respectively. Figure 10 shows representative histological images for the ARDS‐only groups having a score of 2 and 3 for inflammation, respectively. Similarly, Figure 12a–d shows representative histological images and scores for the ITAO, IAAO, ITAS, and ITAS groups, respectively. The pigs in the ARDS‐only group showed a greater histological score for inflammation compared with the control group. With a score of 1.9 ± 0.3 for inflammation, the ARDS‐only group showed a significantly greater value than the control group (*p* < 0.001; Figure 12a). Edema and hemorrhage scores did not show significantly different values among the various groups (Figure 12b,c). The wet/dry ratio between the control and ARDS‐only group showed no significant difference (*p* = 0.707; Figure 13).

## 4 DISCUSSION

Our study developed a novel large animal model of moderate‐to‐severe ARDS with rapid onset and tachycardia. Direct installation of nebulized LPS at a relatively high dose of 4 mg/kg led to a rapid (<4 h) drop in oxygenation to meet the Berlin criterion for moderate disease (PaO_2_/FiO_2_ < 200 mmHg). The mortality rate for this model at 8 h post‐LPS was >25%, indicating that the disease progressed from a moderate stage to a severe stage. This model attempts to mimic rapidly developing ARDS, as may happen in certain critical care situations, such as aspiration pneumonitis (Erdem et al., 2014; Warren et al., 2018). Pompe et al. used a similar approach wherein LPS were nebulized to induce ARDS in mechanically ventilated pigs (Leiphrakpam, Weber, Ogun, & Buesing, 2021). In that study, severe ARDS developed after 166 ± 33 min of LPS nebulization. Chest radiographs of pigs showed bilateral infiltrates. Moreover, signs of pulmonary edema and diffuse neutrophilic alveolitis were evident (Leiphrakpam, Weber, Ogun, & Buesing, 2021).

We utilized this rapid‐onset model of ARDS to compare the efficacy of the IT and IA OMB infusions by studying the blood oxygenation levels and survival rates. The pigs first received a 4 mg/kg dosage of LPS to induce ARDS, the FiO_2_ was titrated down to 21%, and then the pigs either received a 100 ml/kg bolus of OMB via the IA catheter or a 500 ml OMB dose via the IT catheter (or saline controls). The results showed no significant increase in the pigs' SpO_2_ or PaO_2_ values compared with the ARDS‐only and saline treatment groups. These results show that the OMB infusion did not augment the blood oxygenation levels in this ARDS injury model. This result was surprising given prior studies on small animal models (Matsutani et al., 2010; Raghavendran et al., 2011; Swanson & Borden, 2010; Zhao et al., 2015; Buesing et al., 2019) and large animal (Borden, 2021) models of smoke inhalation injury that showed increased blood oxygen and survival following OMB delivery.

Our study also showed that the survival rate of pigs that received saline and OMB infusions was lower than their counterparts from the ARDS‐only group. This result did not change depending on the route of the OMB administration. The lower survival rate suggests a possible OMB infusion‐related injury in the pigs. A possible hypothesis for why rats (but not pigs) experienced O_2_ benefit from OMBs is that a low peritoneal cavity surface area to body mass ratio could have caused insufficient oxygen transfer for a crashing pig. The lower survival of the OMB infusion groups may also have resulted from the pigs in the OMB group's steeper decline in Berlin scores prior to OMB administration than the control or saline groups, which would not be attributable to the OMBs themselves.

Regarding intrathoracic OMB infusion investigation in our moderate ARDS model, we found that the infusion of any significant (>250 ml) volume of OMB or saline into the thoracic cavity resulted in increased mortality in the pigs. This was most likely due to a significant space‐occupying volume of OMB that compromised an already‐failing pulmonary system. In our study, we also observed an increase in the heart rate of the ARDS pigs compared with the control group pigs. Moreover, the heart rates in the experimental groups seemed to increase with duration, though the heart rate did not follow a predictable trend. Follow‐up studies are required to establish the link between OMB or saline infusions and survival outcomes for this and other ARDS models.

A limitation of our study is the small number of animals in the IT administration group. Despite this, we do not recommend the expense of repeating this portion of the study with more animals because we believe the lack of signal of improvement indicates a poor probability of future success using the IT administration route. Other routes, such as colonic delivery, should be investigated as they are less invasive and access more surface area to oxygen transporting vasculature. Though we chose to investigate a rapid‐onset severe ARDS injury model in this work, a less severe and common progression of ARDS in humans takes several weeks to develop and would be an interesting disease model to study in future work.

## 5 CONCLUSIONS

A large pig model of rapid‐onset ARDS with tachycardia was developed and used to study the efficacy of OMB for oxygenation. The OMB administration did not induce a statistically significant or clinically relevant therapeutic effect in this model; instead, both saline and OMB infusion appeared to lower survival rates slightly. While OMB did not prove efficacious in this rapid‐onset ARDS pig model, it may still prove to have therapeutic benefits in slower progressing or less severe ARDS.

## AUTHOR CONTRIBUTIONS

Riaz Ur Rehman Mohammed: Writing, draft preparation, data curation and analysis. Nathaniel T. Zollinger: Design and execution of experiments, writing—reviewing and editing, data curation and analysis. Andrea R. McCain: Design and execution of experiments, data analysis. Roser Romaguera‐Matas: Design and execution of experiments, data analysis. Seth P. Harris: Histological analysis, reviewing and editing. Keely L. Buesing: Study conception, design of experiments, perform experiment surgeries, mentor and advisor for vet staff procedures, reviewing and editing. Mark A. Borden: Design of experiments, data analysis, reviewing and editing. Benjamin S. Terry: Study conception, supervision, design of experiments, analysis, reviewing and editing.

## FUNDING INFORMATION

The project was funded by the Department of Defense—Offutt Air Force Base STRATCOM award number FA460018D9001; FU911.

## CONFLICT OF INTEREST

None.

## ETHICS STATEMENT

All animal experiments for this study were performed ethically per the University of Nebraska‐Lincoln Animal Care and Use Committee (IACUC) and the Animal Care and Use Review Office (ACURO).
